# Drought response strategies of Arbequina, Arbosana and Koroneiki olive cultivars revealed by ring growth, wood anatomy and δ¹³C dynamics

**DOI:** 10.3389/fpls.2025.1623127

**Published:** 2025-09-04

**Authors:** Silvia Portarena, Matthias Saurer, Enrico Brugnoli, Daniela Farinelli, Paolo Cherubini

**Affiliations:** ^1^ Institute of Research on Terrestrial Ecosystems (IRET), National Research Council (CNR), Porano, Italy; ^2^ National Biodiversity Future Center, Palermo, Italy; ^3^ Forest and Soil Ecology, Swiss Federal Institute for Forest, Snow and Landscape Research WSL, Birmensdorf, Switzerland; ^4^ Department of Agricultural, Food and Environmental Sciences (DSA3), University of Perugia, Perugia, Italy; ^5^ Faculty of Forestry, University of British Columbia, Vancouver, BC, Canada

**Keywords:** olive ring boundaries, dendrochronology, ecophysiology, xylem anatomy, climate adaptation, Mediterranean agriculture

## Abstract

The olive tree (*Olea europaea* L.), a key crop in Mediterranean climates, is increasingly affected by climate variability. Over the last several decades, the Umbria region of central Italy, with its long-standing olive-growing tradition, has experienced a rise in extreme summer droughts, severely impacting water availability. This makes it an ideal case study for investigating olive tree responses to climatic stress. In this study, we examined the adaptive strategies of three economically important cultivars – Arbequina, Arbosana, and Koroneiki – grown as mature trees (7 years old) between 2020 and 2023. We combined dendrochronological techniques, wood anatomical analyses, and intra-seasonal δ¹³C profiling to assess growth dynamics, structural adjustments, and eco-physiological responses across four growing seasons. Our results revealed distinct cultivar-specific strategies in response to climate variation. In Arbequina and Arbosana, δ¹³C values showed significant correlations with current-year spring and summer climate conditions, as well as with conditions during the preceding winter, reflecting a more isohydric behavior. In contrast, Koroneiki exhibited a more anisohydric strategy: its δ¹³C values were primarily influenced by precipitation from the previous winter, indicating a reliance on stored carbon reserves to support early-season growth. Wood anatomical traits further supported these differences. Koroneiki exhibited higher vessel density and a greater proportion of lumen area, traits that enhance water transport efficiency. It also achieved the highest stem basal area and fruit production among the three cultivars, reaching 10.2 kg/tree in 2023. These characteristics highlight Koroneiki’s potential as a drought-resilient cultivar suited for future orchard designs in Mediterranean regions increasingly affected by heat and water stress.

## 1 Introduction

The olive tree (*Olea europaea* L.) has traditionally been valued for its resilience and adaptability to dry environments. However, changing climate conditions – particularly in the Mediterranean basin, the crop’s primary cultivation area – are posing significant challenges. Rising temperatures, prolonged droughts, and irregular heavy rainfall events are increasingly compromising crop health and productivity (Rico et al., 2024). In Italy, where most olive trees are cultivated under rainfed conditions, there is a growing demand for innovative management strategies to enhance water stress tolerance (Branquinho et al., 2021; Famiani et al., 2022). Olive oil, a cornerstone of the Mediterranean diet known for its beneficial fatty acids and antioxidants (Cinosi et al., 2024; Portarena et al., 2023), holds substantial economic importance across several Mediterranean regions (Marchioni et al., 2024). As a result, recent studies emphasize the need for adaptive agricultural practices that reduce water demands while maintaining both productivity and quality (Adi et al., 2025; Famiani et al., 2022; Fernández et al., 2013). Understanding how different olive cultivars respond to environmental changes is thus essential for ensuring the sustainable management and long-term resilience of olive production systems (Rossi et al., 2013; Bacelar et al., 2009; Brito et al., 2019).

Dendroecological methods provide powerful tools for analyzing climate-growth relationships in trees, offering insights into physiological responses to environmental changes (Cherubini et al., 2021; Fonti et al., 2010; Siegwolf et al., 2022). Variations in tree-ring width and wood anatomical traits reflect adaptive structural adjustments that optimize support, storage, and transport of water and nutrients under changing environmental conditions and phylogenetic constraints (Gričar and Eler, 2025; Balzano et al., 2020; De Micco et al., 2019). Differences in xylem hydraulic architecture reveal a cultivar's plasticity in response to environmental variability (Damiano et al., 2023; Fonti et al., 2010; Sabella et al., 2020). In parallel, stable carbon isotopic composition (δ^13^C) in tree rings allows retrospective analysis of climatic impacts on growth and key physiological traits, such as stomatal conductance and photosynthetic performance (Portarena et al., 2022; Portarena et al., 2024; Altieri et al., 2015).

During dry Mediterranean summers, trees often reduce stomatal conductance to limit water loss, leading to decreased photosynthetic rates. These physiological changes affect carbon isotope fractionation during CO_2_ uptake and fixation, thereby altering the δ^13^C composition of organic matter (Brugnoli and Farquhar, 2000). Consequently, both inter- and intra-annual climate variations are recorded in δ^13^C patterns along tree rings (Monson et al., 2018). However, δ^13^C analysis in olive tree wood remains relatively unexplored (Rossi et al., 2013; Sabella et al., 2020; Portarena et al., 2024), largely because of the difficulty in accurately identifying annual tree-ring boundaries. This challenge is exacerbated by mild winters, which prolong the growing season, and by drought-induced false ring formation (Cherubini et al., 2003; Cherubini et al., 2013; Battipaglia et al., 2016).

To address this gap, we integrated dendrochronology, wood anatomy, and intra-seasonal δ^13^C profiling to investigate the retrospective growth and ecophysiological dynamics of olive trees. Focusing on three economically important olive cultivars – Arbequina, Arbosana, and Koroneiki – grown in an experimental orchard in Umbria, central Italy, we analyzed their responses over the 2020–2023 growing seasons. Our aim was to evaluate how physiological adjustments to climate variability are reflected in growth patterns and yield. We hypothesized that these cultivars adapt their traits to environmental variations through distinct strategies, corresponding to different ecophysiological types.

## 2 Materials and methods

### 2.1 Orchard conditions, cultivars and fruit production

The study was conducted between 2017 and 2023 in a rainfed, high-density olive orchard located in central Italy (42°59′23″ N, 12°41′47″ E, 260 m a.s.l.). The orchard covers 10,000 m² and is planted at a density of 1,000 trees per hectare, comprising three international olive cultivars: Arbequina, Arbosana, and Koroneiki.

Arbequina and Arbosana, both native to Catalonia (northeastern Spain), share several traits, including compact growth habits, small fruit size, and high oil content, making them particularly suitable for dense planting and mechanical harvesting (Vidal et al., 2019). Koroneiki, a cultivar native to Greece, is more vigorous, producing small fruits with high oil content, and is highly valued in its region of origin (Vidal et al., 2019).

The trees were transplanted into the orchard in autumn 2016 at the age of eight months, with an average height of approximately 60 cm. They were trained using a central leader system with multiple lateral branches and spaced at 2 m × 5 m to form hedgerows. Sampling was conducted within a defined, homogeneous 500 m² area to minimize environmental variability among cultivars. Within this area, three trees per cultivar were randomly selected to avoid potential bias.

The soil has a medium-clay texture with the following characteristics: pH 7.9 ± 0.1, organic matter content 1.4 ± 0.1 (%), active limestone 3.6 ± 0.1 (%), cation exchange capacity 25.6 ± 0.5 (meq/100 g), exchangeable potassium: 273 ± 43 (mg/kg), and assimilable phosphorus 31.9 ± 2.5 (mg/kg) (Famiani et al., 2022).

Temperature and precipitation data were obtained from a meteorological station adjacent to the experimental field. Monthly air temperatures and rainfall amounts were recorded from January 1^st^ to December 31^st^ for each year between 2020 and 2023.

At the beginning of each November, total fruit production per tree was determined by manually harvesting olives from three trees per cultivar. Fruit production started from 2019 onward, when the trees entered their mature productive phase.

### 2.2 Tree-ring sample collection and preparation

The same nine trees used for fruit production measurements (height: 3 ± 0.3 m; stem diameter: 11 ± 0.2 cm; mean ± standard deviation) were also used for wood sampling. One disk was collected from the main branch of each tree at 1.2 m above the ground in October 2023. Disks were air-dried and polished using progressively finer sandpaper to enhance cell visibility and delineate tree-ring boundaries.

### 2.3 Tree-ring width and anatomical analysis

Each sample was digitalized using the Skippy image-capturing system (WSL, 2020; https://www.wsl.ch/en/services-produkte/skippy/) equipped with a Sony Alpha 7R IV (61-megapixel) camera that captures high-resolution (6000 dpi) pictures every 6 mm. Images were stitched together using PTGui and cropped to reduce file size.

Ring width (RW) was measured using the program CooRecorder (Cybis Elektronik & Data AB, Saltsjöbaden, Sweden). Annual rings were manually marked, and by assigning the outermost ring to the most recent year, all rings were automatically dated. Due to asymmetric cambial growth, RW was measured along four radii per disk, and the four series were averaged after visual cross-dating using common marker years and patterns (Stokes and Smiley, 1968). Cross-dating accuracy and measurement consistency were validated with the dplR package (Bunn, 2008) in R.

Anatomical measurements followed the protocol of Von Arx et al. (2016). Each disk was split into 3–4 cm long (branch diameter) and 1 cm width pieces. Transverse microsections (15 μm thick) were cut with a WSL lab microtome (Gärtner et al., 2015), stained with a solution of safranin (1%) and astra-blue (0.5%) to increase visual contrast, dehydrated with ethanol, mounted on glass slides with Eukitt UV (Bio Optica), and stabilized under UV light for about 2 minutes.

High-resolution micro-photos were taken with a Zeiss Slide Scanner Axio Z1 at 100x magnification. Vessel lumen area (VLA), vessel density (VD), and vessel lumen fraction (VLF, calculated as the ratio between the total vessel lumen area within a specific section of the ring and the area of that same section) were measured for earlywood (EW) and latewood (LW) in each ring using WinCELL (ver. 2019a, Regent Instruments Canada). Anatomical analyses have been focused on the period starting in 2020, when trees reached physiological and productive maturity.

### 2.4 Tree-ring δ^13^C analyses and standardization of the δ^13^C time series

Stable carbon isotope ratios (δ^13^C) were measured by laser-ablation coupled with isotope ratio mass spectrometry (LA-IRMS) following Saurer et al. (2023). The same wood samples used for anatomical measurements (three replicates per cultivar) were prepared with a microtome to produce a flat surface, and placed in the sample chamber. A series of shots (100 μm diameter, 300 μm distance apart) were fired across rings covering the 2020–2023 growth periods. Wood fragments were ablated using a UV-laser (Teledyne LSX-213 G2+, Nd : YAG Laser; wavelength 213 nm); the resulting dust and gas were combusted to CO_2_, and the ^13^C/^12^C ratios were measured with IRMS (HS2022, Sercon, Crewe, UK). Calibration was performed using two cellulose reference materials (Saurer et al., 2023). As for anatomical analyses, δ¹³C analyses were also focused on the period starting in 2020, when the trees reached physiological and productive maturity.

**Figure 1:**
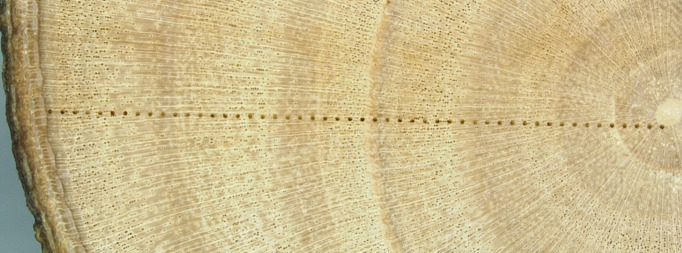
Laser shots (100 μm) spaced 300 μm apart in a magnified cross-section of an olive tree branch from the cultivar Arbosana.

Annual variation in tree-ring width resulted in differing numbers of δ^13^C measurements per year across samples (**Figure 1**).

To allow comparisons, the number of analyses was standardized by matching the tree with the fewest measurements for EW and LW in each year. Measurements from other trees were averaged as necessary to create uniform time series with the same number of points across trees and years.

### 2.5 Statistical analysis

Ring width (RW) data were converted into basal area increment (BAI) using the formula:


(1) $$\text{BAI}=\text{π}({\text{r}}_{\text{n}}^{2}–{\text{r}}_{\text{n}-1}^{2})$$

where r is the ring radius and n is the year of formation.

For RW, BAI, VLA, VD, VLF, and δ^13^C, mean values and standard errors (SE) were calculated from three plants per cultivar. To assess isotopic variability, standard deviations of δ¹³C values were calculated across the entire study period.

Data normality was tested with the Shapiro-Wilk test. Pearson correlation coefficients were computed between (i) δ¹³C values and anatomical data and (ii) climatic variables (cumulative precipitation and mean temperature during October-February, March-May, and June-August) and RW, BAI, and δ^13^C (annual maximum and mean). These temporal divisions allowed us to better capture and interpret the climate-growth relationships during the most influential periods of the annual cycle. The October–February window, covering the previous year’s late autumn and winter, is relevant for processes such as reserve accumulation and bud development during dormancy, which influence growth at the start of the following season. March to May corresponds to early vegetative activity, budding, flowering, and initial fruit set phases that are particularly sensitive to temperature and water availability. June to August represents the peak of summer drought in Mediterranean climates, when water and heat stress is most intense and fruit growth occurs.

A repeated measures ANOVA evaluated the effects of interannual variation (YEAR, random effect), cultivar (CV, fixed effect), and their interaction on RW, BAI, and fruit yield. For vessel traits (VA, VD, VLF), wood type (EW, LW; fixed factor), was also included. Sum of squares (SS), mean square (MS), and F-values were computed, with significance assessed at p < 0.05.

All statistical analyses were performed using Statistica v10 (Stat-Soft Italia srl, Padua, Italy) and R (R Development Core Team 2018, Vienna, Austria).

## 3 Results

### 3.1 Weather conditions

**Figure 2:**
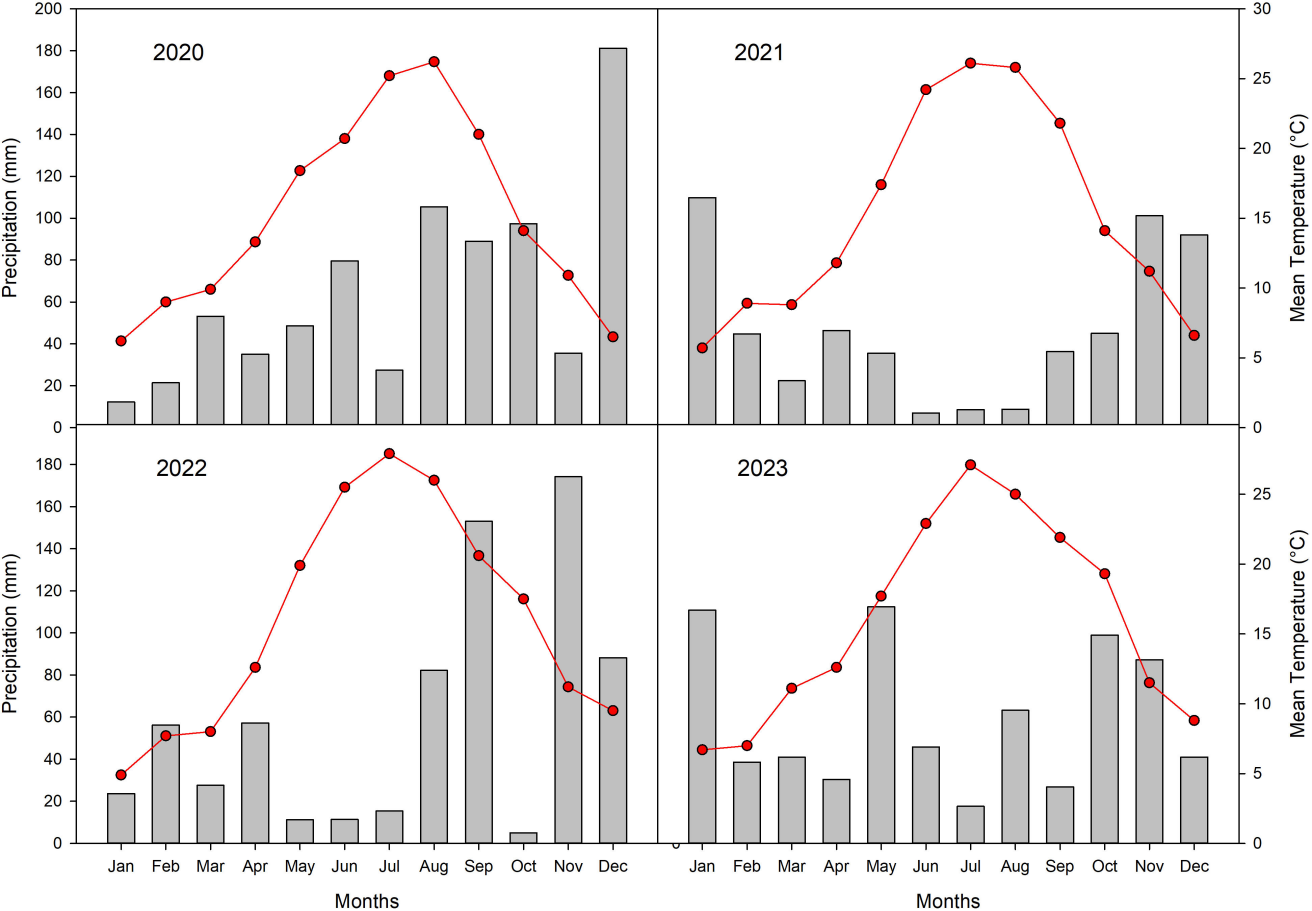
Climatic data (cumulated monthly rainfall, grey bars and mean monthly temperatures, red line) recorded in the experimental orchard from 2020 to 2023.

Annual rainfall varied throughout the study period, ranging from a high of 786 mm in 2020 to a low of 558 mm in 2021, with intermediate values recorded in 2022 (705 mm) and 2023 (713 mm) (**Figure 2**).

Precipitation during the summer months (June-August) showed substantial interannual variability. In 2020, summer rainfall was relatively high at 212.4 mm. In contrast, 2021 experienced a pronounced drought, with only 24.4 mm of rainfall recorded. Summer precipitation partially recovered in subsequent years, reaching 109 mm in 2022 and 126.6 mm in 2023.

Temperature patterns displayed a clear seasonal trend, with increases from January through July/August, followed by declines towards December. Summer temperatures remained consistently high across the study period. In 2020, mean monthly temperatures rose from 20.7°C in June to a peak of 25.2°C in July. Similarly, in 2021 and 2022, June temperatures averaged 24.2°C and 25.5°C, respectively, with July temperatures reaching 26.1°C and 27.9°C, respectively. In 2023, June, July, and August temperatures averaged 22.9°C, 27.2°C, and 25°C, respectively, confirming persistently warm summer conditions.

### 3.2 Tree-ring width analysis

The repeated measures ANOVA indicated a significant effect of year on both RW and BAI (**Equation 1**). Cultivar had a significant effect on BAI but not on RW, while no significant interaction between year and cultivar was observed for either variable (**Table 1**).

**Table 1:** Repeated measures ANOVA for ring width (RW) and basal area increment (BAI) across years (random effect) and cultivars (fixed effect).

	RW				BAI			
Effect	SS	MS	F	p	SS	MS	F	p
Year	**12.3**	**2.1**	**2.9**	**0.018**	**386740**	**64457**	**26.5**	**0.000**
Cultivar	4.1	2.1	2.9	0.063	**43910**	**21955**	**9.0**	**0.001**
Year*Cultivar	12.7	1.1	1.5	0.161	25802	2150	0.9	0.570

Significant p-values (< 0.05) are shown in bold.

The symbol * indicates the interaction between the effects (e.g. Year × Cultivar).

**Figure 3:**
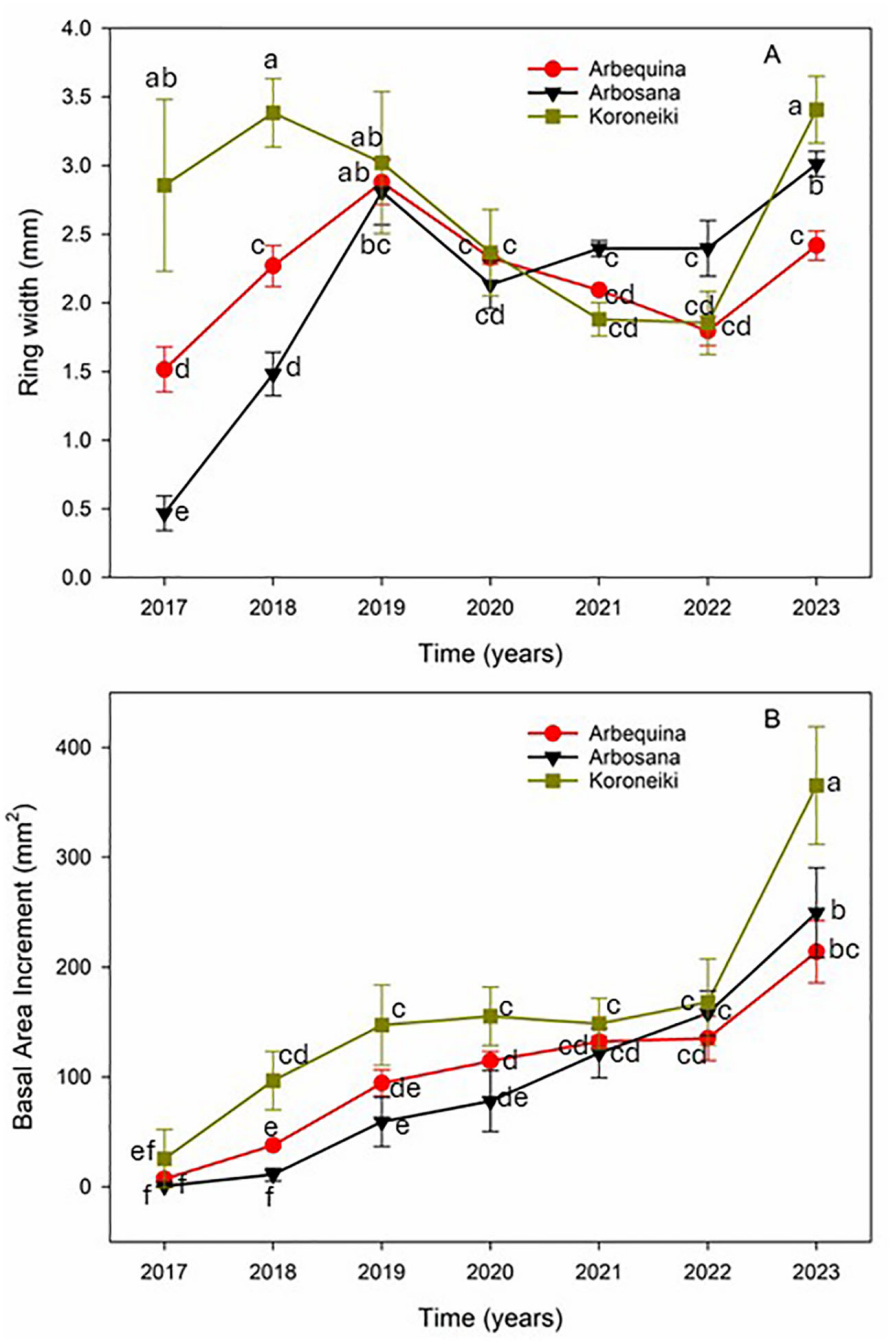
Components of radial growth in olive trees from the cultivars Arbequina (red circles), Arbosana (black triangles), Koroneiki (green squares). **(A)** Inter-annual variability in ring width (RW) and **(B)** Basal area increment (BAI). Values represent means ± standard errors. Statistical differences were assessed using Fisher’s *post hoc* multiple comparison test. Different letters indicate statistically significant differences at p < 0.05.

The sampled branches recorded a growth chronology covering the seven-year period from 2017 to 2023 (**Figure 3**). Growth patterns exhibited a coherent signal across cultivars, with the expressed population signal (EPS) exceeding the critical threshold of 0.85, indicating a reliable cross dating and a strong common environmental influence on growth.

Ring width (RW) showed significant variability from 2020 to 2023 (**Figure 3**). Across all cultivars, BAI increased substantially over the period from 2017 to 2023. Koroneiki consistently exhibited the highest BAI, starting at 25.64 mm² in 2017 and reaching 365.33 mm² by 2023 (**Figure 3**).

### 3.3 Tree-ring anatomical analyses

The repeated measures ANOVA revealed several significant effects (**Table 2**). Year significantly influenced vessel area (VA), while cultivar had a significant effect on vessel density (VD) and vessel lumen fraction (VLF). Wood type (earlywood [EW] *vs*. latewood [LW]) significantly influenced all three anatomical traits. Additionally, the year*cultivar interaction was significant for both VA and VD, while interactions between year*wood and year*cultivar*wood were significant for VA only.

**Table 2:** Repeated measures ANOVA of wood anatomical parameters (vessel lumen area, VLA; vessel density, VD; vessel lumen fraction, VLF) across years (random effect), cultivars (fixed effect), and wood type (fixed effect).

	VLA				VD				VLF			
Effect	SS	MS	F	p	SS	MS	F	p	SS	MS	F	p
YEAR	**43687**	**14562**	**3.5**	**0.023**	4640	1547	1.6	0.193	0.00	0.00	0.05	0.985
CULTIVAR (CV)	22643	11321	2.7	0.076	**130239**	**65120**	**69.1**	**0.000**	**0.04**	**0.02**	**12.88**	**0.000**
WOOD	**1152071**	**1152071**	**277.1**	**0.000**	**72381**	**72381**	**76.9**	**0.000**	**0.08**	**0.08**	**46.96**	**0.000**
YEAR*CV	**171856**	**28643**	**6.9**	**0.000**	**13649**	**2275**	**2.4**	**0.041**	0.00	0.00	0.29	0.939
YEAR*WOOD	**200103**	**66701**	**16.0**	**0.000**	3900	1300	1.4	0.261	0.00	0.00	0.46	0.713
CV*WOOD	24395	12197	2.9	0.063	5454	2727	2.9	0.065	0.01	0.00	2.48	0.095
YEAR*CV*WOOD	**50713**	**10143**	**2.4**	**0.048**	1905	381	0.4	0.843	0.00	0.00	0.09	0.993

Significant p-values (< 0.05) are shown in bold.

The symbol * indicates the interaction between the effects.

**Figure 4:**
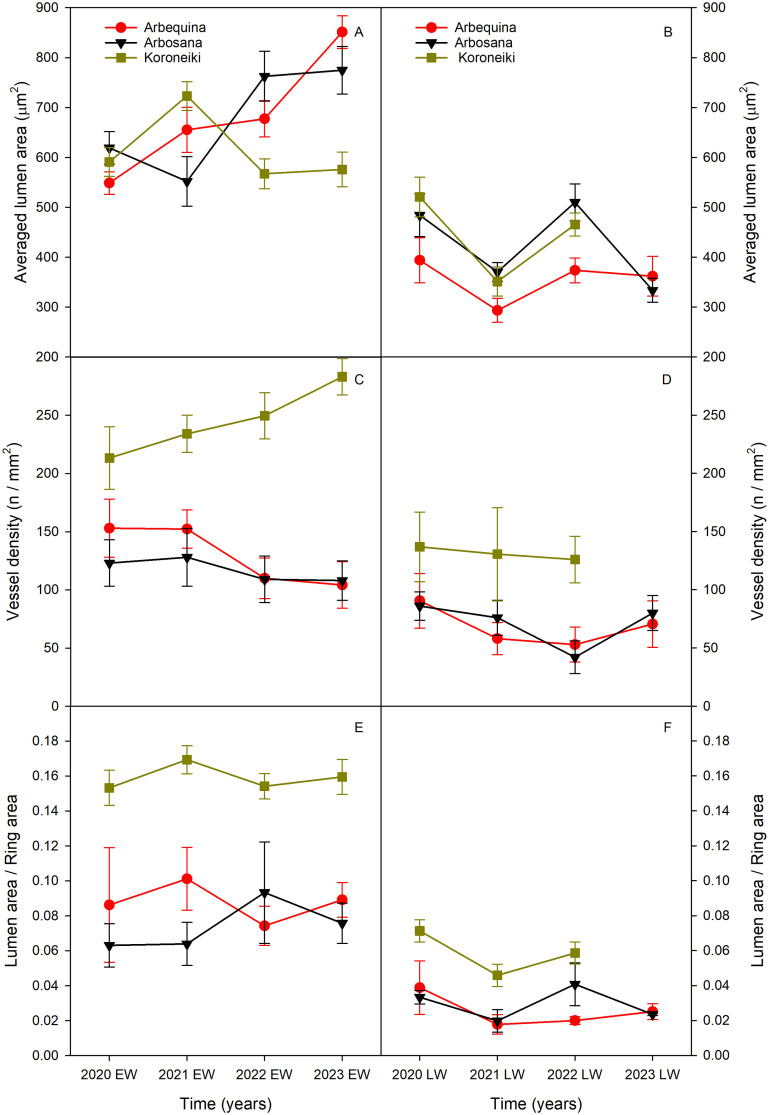
Variation in (a) vessel lumen area (VLA; **A, B)**, (b) vessel density (VD; **C, D)**, and (c) vessel lumen fraction (VLF, **E, F)** along ring width from earlywood (EW, left column) to latewood (LW; right column) over the period 2020-2023. Values are expressed as mean ± standard error. Cultivars are represented by different symbols: red circles for Arbequina, black triangles for Arbosana, green squares for Koroneiki. Note: 2023 latewood data for the Koroneiki cultivar are unavailable due to sampling limitations.

Across 2020-2023, all anatomical traits (VLA, VD, VLF) were significantly lower in latewood compared to earlywood (**Figure 4**).

Koroneiki exhibited the highest VD, increasing from 137 vessels/mm² in 2020 to 283 vessels/mm² by 2023. In contrast, Arbequina and Arbosana displayed lower and more variable VD values. Arbequina started with approximately 153 vessels/mm² in 2020, gradually declining to about 104 vessels/mm² by 2023. Arbosana followed similar trend, with earlywood VD around 100 vessels/mm² in 2020, decreasing to about 83 vessels/mm² by 2023.

VLF patterns indicated that Koroneiki maintained higher values in both earlywood (>0.150) and latewood (>0.046) compared to Arbequina and Arbosana. The latter two cultivars showed lower VLF values, fluctuating between 0.06 and 0.10 in earlywood and between 0.01 and 0.04 in latewood.

### 3.4 Tree-ring δ^13^C analyses

**Figure 5:**
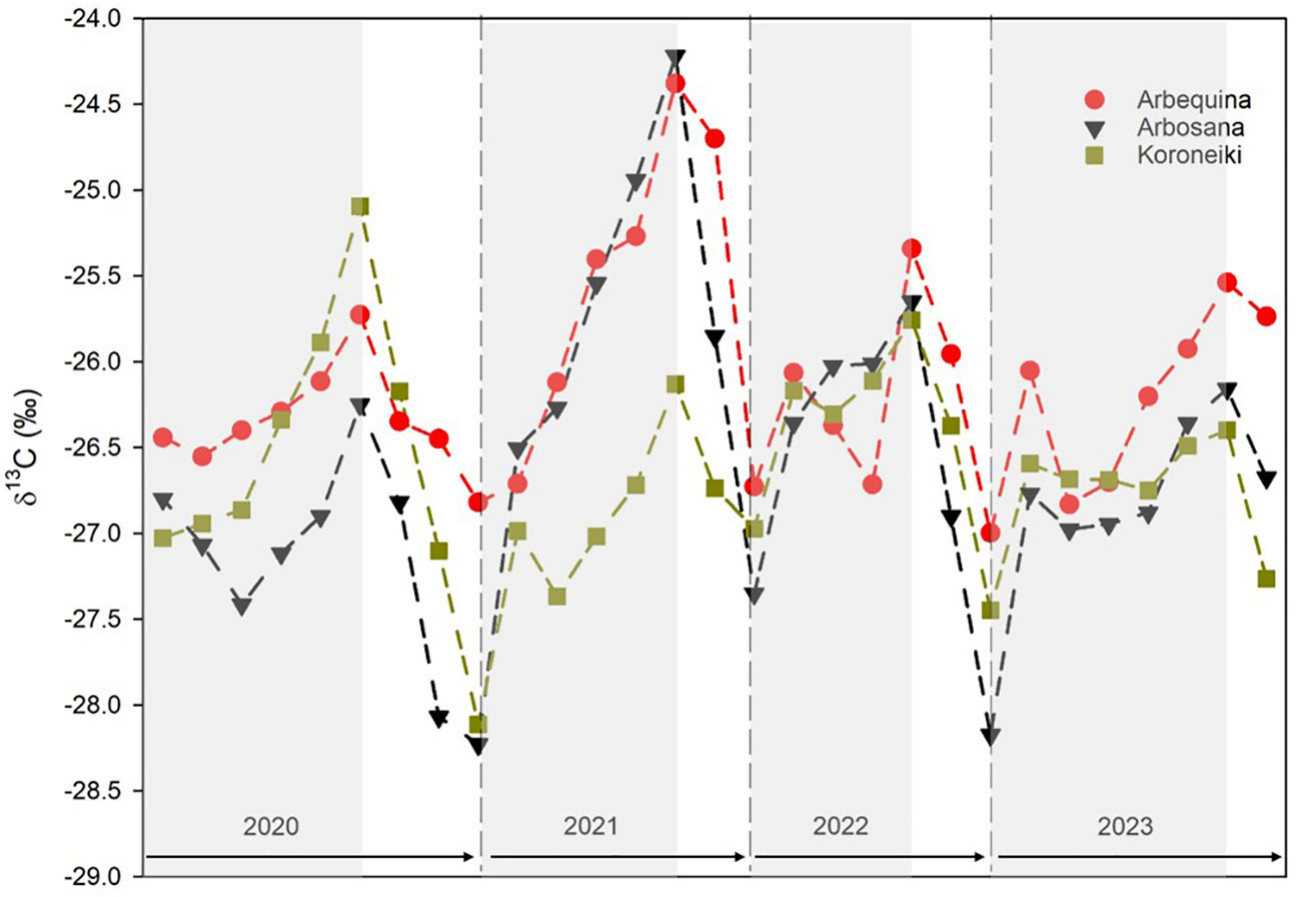
Intra-annual δ^13^C values across tree rings of *Olea europaea* L. cultivars measured by LA-IRMS. Cultivars are represented by different symbols: red circles for Arbequina, black triangles for Arbosana, green squares for Koroneiki. Ring boundaries are indicated, with earlywood (EW) shown on a shaded background and latewood (LW) on a white background. Values are expressed as cultivar means; standard error bars were omitted for clarity.

The intra-annual δ^13^C patterns varied considerably across the 2020–2023 period, spanning a range of more than 4‰ (**Figure 5**).

All three cultivars exhibited similar fluctuations, with values increasing in earlywood (EW) and decreasing in latewood (LW). The most negative δ^13^C values occurred near the transition between LW and EW, where annual ring formation likely commenced, while the highest δ^13^C enrichments were found mid-ring, between EW and LW. However, the magnitude of δ^13^C variation from the start of EW to the end of LW differed among cultivars and years.

Arbosana showed the largest δ^13^C variation (4‰) with a standard deviation of 0.88‰, compared to 0.64‰ for Arbequina and 0.59‰ for Koroneiki. In 2020, Arbosana’s δ^13^C displayed a slight increase followed by a sharp decline to its most negative mean value (-28.3‰). In 2021, Arbosana exhibited the largest intra-annual δ^13^C increase and range. In 2022, δ^13^C initially rose before decreasing to values similar to the previous year. In 2023, values again slightly increased with some fluctuations.

Arbequina displayed more consistent δ^13^C fluctuations across the study period, with less pronounced variability compared to Arbosana. A moderate increase and decrease was noted in 2020; 2021 saw a significant rise to the highest δ^13^C peak recorded. In 2022 and 2023, δ^13^C increased again, though fluctuations were mostly restricted to the rising phases.

Koroneiki showed the greatest δ^13^C variation in 2020 but was relatively stable in the subsequent years, with only minor fluctuations during the EW phases.

Importantly, LW δ^13^C data for 2023 were incomplete because sampling occurred at the end of October, before LW development was complete. As a result, 2023 δ^13^C values did not reach the more negative levels observed in previous years.

**Figure 6:**
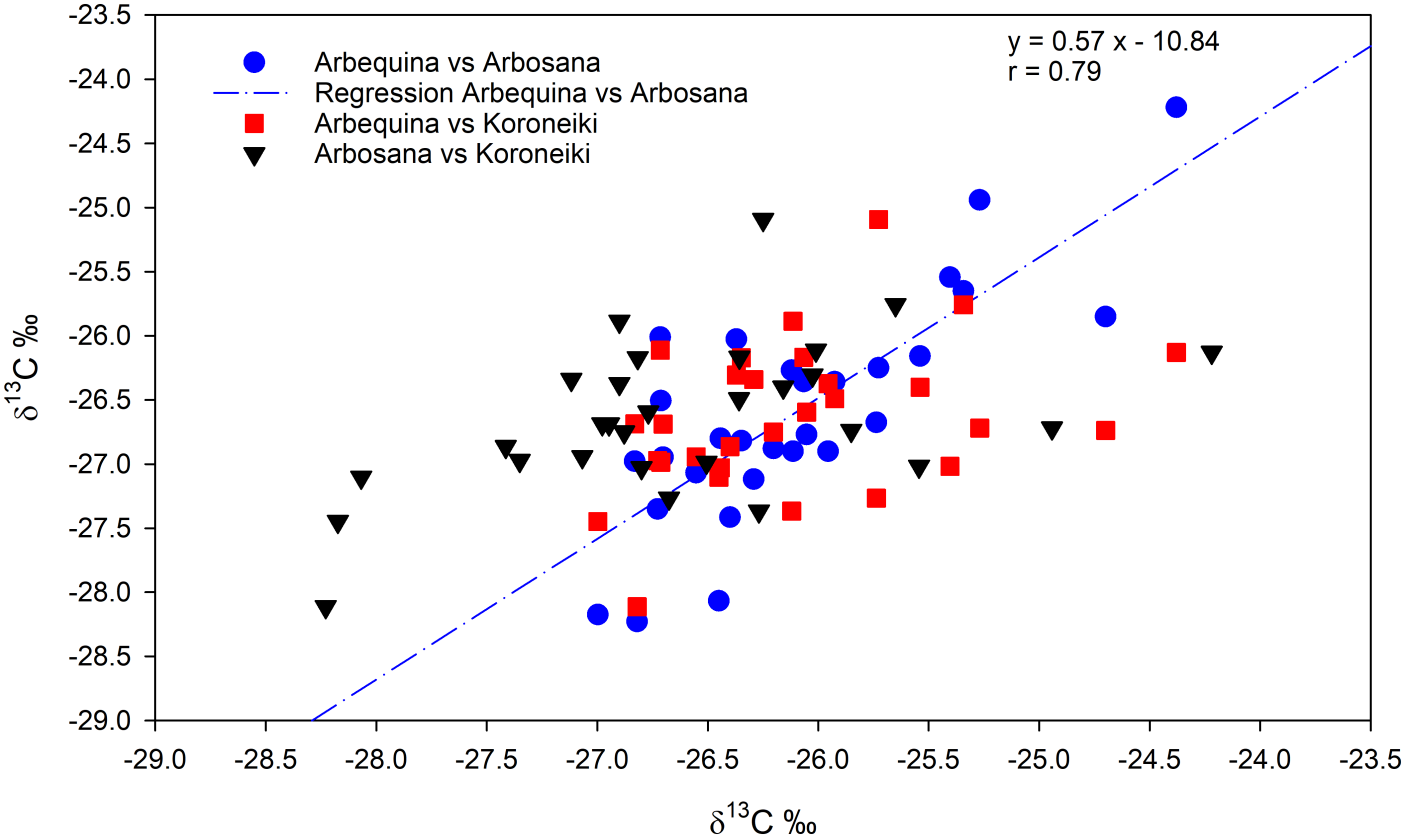
Pairwise correlations of intra-annual δ^13^C values across the tree rings of the three cultivarsover the full study period. Arbequina *vs* Arbosana is represented by blu circles, Arbequina *vs* Koroneiki is represented by red squares, and Arbosana *vs* Koroneiki is represented by black trangles. The regression line and equation are shown for the significant correlation between Arbosana and Arbequina.

A significant negative correlation between annual δ¹³C and anatomical traits was observed. Specifically, across all cultivars, annual δ¹³C was negatively correlated with vessel density (VD), both in EW (r = –0.42, p = 0.015) and in latewood (r = –0.47, p = 0.006). Comparing cultivars, a significant and positive correlation was found between the δ^13^C values of Arbosana and Arbequina over the entire period. In contrast, no significant correlations emerged between Koroneiki and the other cultivars (**Figure 6**).

### 3.5 Climate correlations and fruit production

**Table 3** presents the correlations between ring growth (RW and BAI), δ^13^C values, and climatic variables for each cultivar. As no distinct climate signals were associated with EW and LW anatomical traits, their correlation results were excluded.

**Table 3:** Pearson correlation coefficients between climatic factors (seasonal precipitation, prec; temperature, T) and ring width (RW), basal area increment (BAI), maximum δ^13^C, and annual mean δ^13^C.

	Arbequina				Arbosana				Koroneiki			
Climate factor	RW	BAI	δ^13^C Max	Annual δ ^13^C	RW	BAI	δ ^13^C Max	Annual δ ^13^C	RW	BAI	δ ^13^C Max	Annual δ ^13^C
prec Oct-Feb	0.12	0.36	0.51	0.14	0.32	0.28	0.57	0.15	0.1	0.25	**-0.67**	**-0.62**
prec Mar-May	**0.64**	**0.65**	-0.35	**-0.75**	0.44	0.46	-0.49	**-0.72**	**0.68**	**0.74**	-0.26	-0.22
prec Jun-Aug	0.32	-0.08	**-0.61**	-0.58	-0.14	-0.14	**-0.73**	-0.53	0.24	0.08	0.55	0.45
T Oct-Feb	**0.68**	0.23	-0.43	**-0.81**	0.09	0.02	-0.55	**-0.7**	0.49	0.37	0.21	0.07
T Mar-May	0.32	0.45	**-0.66**	**-0.6**	0.11	0.19	**-0.81**	**-0.62**	0.41	0.37	0.28	0.32
T Jun-Aug	**-0.63**	0.05	0.23	**0.68**	0.1	0.26	0.3	0.5	-0.29	-0.08	-0.32	-0.07

Significant values (p<0.05) are highlighted in bold.

The radial growth (RW) of Arbequina and Koroneiki was significantly and positively correlated with spring precipitation. Although Arbosana also showed a positive trend, it was not significant. Arbequina’s RW was also positively correlated with the previous year’s winter temperatures and negatively correlated with summer temperatures. In contrast, Arbosana and Koroneiki radial growth showed no significant correlation with temperature variables.

Both Arbequina and Arbosana displayed negative correlations between annual δ^13^C and spring precipitation, previous winter temperatures, and spring temperatures, and positive correlations with summer temperatures. Maximum δ^13^C values were negatively associated with summer precipitation and spring temperatures.

Koroneiki’s δ^13^C dynamics differed: both its annual and maximum δ^13^C values were significantly correlated with precipitation during the previous winter, but not with spring and summer climate variables of the current year.

Fruit production, expressed as olive yield (kg/tree), is presented in **Table 4**.

**Table 4:** Fruit yield (kg/tree) per cultivar from 2019 to 2023.

Cultivar	Yield 2019 (kg/tree)	Yield 2020 (kg/tree)	Yield 2021 (kg/tree)	Yield 2022 (kg/tree)	Yield 2023 (kg/tree)
Arbequina	0.73 f	4.68 d	0.00	6.20 b	6.78 b
Arbosana	0.91 f	3.86 d	3.57 d	7.13 b	3.82 d
Koroneiki	0.35 g	2.81 e	6.09 c	5.74 c	10.21 a

Statistical differences were assessed using Fisher’s *post hoc* multiple comparison test. Different letters indicate significant differences among cultivar × year means (p<0.05).

Arbequina exhibited a marked increase in fruit production from 2019 to 2020, no fruit production in 2021, and consistent increases in 2022 and 2023, maintaining relatively high yields during the last two years. For Arbosana, fruit yield remained similar across 2020, 2021 and 2023, with higher fruit production observed in 2022, according to **Table 4** Koroneiki, starting with the lowest yield in 2019, displayed steady year-on-year increases, achieving the highest yield in 2023, suggesting a strong upward trend in productivity.

## 4 Discussion

This study examined three olive cultivars in central Italy, revealing distinct strategies in response to seasonal climate variations affecting fruit production. Patterns in ring growth indicated adaptive responses to environmental changes, particularly pronounced in the Koroneiki cultivar. A significant positive correlation between RW and spring precipitation, observed in Koroneiki and Arbequina, highlighted the critical role of water availability during spring. Reduced RW in 2021 and 2022 in these cultivars likely resulted from decreased spring rainfall. In contrast, the absence of a significant relationship between RW and climate variables in Arbosana suggests that its cambial activity may be less sensitive to seasonal changes.

Moreover, a negative correlation between ring growth and summer temperatures in Arbequina suggests reduced cell wall formation during summer xylogenesis. Elevated temperatures may exacerbate water deficits through increased evapotranspiration and soil moisture loss, limiting photosynthesis and carbohydrate supply for cambial growth (Battipaglia et al., 2010; Balzano et al., 2020).

Anatomical analyses revealed consistent features across cultivars: earlywood (EW) exhibited larger cells and higher density compared to latewood (LW), reflecting seasonal phases of xylogenesis (Gričar and Eler, 2025; Von Arx et al., 2016; Zhirnova et al., 2021). While EW structure is primarily habitat-driven in evergreen species, LW characteristics depend more on species-specific and seasonal factors (Zhirnova et al., 2021). Despite climatic variations, similarities in vessel lumen area (VLA) across cultivars suggest that genetic factors may also influence cell enlargement (Fonti et al., 2010; Castagneri et al., 2017). Notably, Koroneiki displayed higher vessel density (VD) and vessel lumen fraction (VLF), potentially supporting greater hydraulic capacity under varying climatic conditions. Enhanced water transport efficiency could help maintain stomatal conductance, growth and fruit production, especially under optimal conditions (Piermattei et al., 2020; Attia et al., 2015).

Variation in xylem anatomy among cultivars likely influences stomatal regulation and protects against hydraulic failure (Sabella et al., 2019).

The δ^13^C variability in wood rings revealed distinct seasonal patterns among cultivars, reflecting physiological responses to environmental conditions. Across all cultivars, EW consistently exhibited higher δ^13^C values than LW, corresponding to spring and autumn wood formation, respectively, with LW formation coinciding with the end of fruit development (Portarena et al., 2024). Each year, a δ^13^C maximum was observed near the EW-LW transition, particularly under low summer precipitation for Arbosana and Arbequina (**Figure 5**). High-resolution δ^13^C analyses effectively captured these seasonal dynamics.

From spring to summer, rising temperatures and decreasing rainfall (**Figure 2**) likely reduced the intercellular to atmospheric CO_2_ ratio (Ci/Ca), elevating δ^13^C values due to partial stomatal closure and reduced photosynthetic CO_2_ uptake (Brugnoli and Farquhar, 2000). Conversely, wetter autumn and winter conditions increased Ci/Ca, resulting in lower δ^13^C values during LW formation (Brugnoli and Farquhar, 2000; Farquhar et al., 1989).

Interestingly, in 2021, both Arbosana and Arbequina exhibited particularly high δ¹³C values. This isotopic signal coincided with markedly low fruit production in both cultivars - complete absence in Arbequina and reduced yield in Arbosana - suggesting that diminished sink demand may have influenced carbon allocation dynamics. During OFF years or under low crop load, reduced translocation of photoassimilates to fruits can lead to feedback inhibition of photosynthesis, stomatal closure, and increasing ¹³C accumulation in vegetative organs including annual branches (Ding et al., 2017).

Interestingly, Koroneiki showed less δ^13^C variability, suggesting greater drought resilience and a more conservative water-use strategy. This stability may be supported by increased VD, allowing for moderated stomatal conductance and photosynthetic rates, as suggested by the observed negative correlation between annual δ¹³C and VD. Future research could explore whether differences in root expansion and root-to-shoot ratios among cultivars contribute to variations in water-use efficiency.

The occasional depletion in δ^13^C just before reaching annual maxima may reflect short-term drought, high temperatures, or elevated vapor pressure deficits, all of which can temporarily raise Ci/Ca and lower δ¹³C (Farquhar et al., 1989). Additional factors, such as carbon isotope partitioning during fruit lignification (Badeck et al., 2005) and vegetative-to-flowering transitions, may also influence δ^13^C values (Portarena et al., 2024).

Correlations between δ^13^C and seasonal climate data emphasize the link between water availability during wood formation and seasonal carbon allocation dynamics (Gessler and Treydte, 2016; Kagawa et al., 2006; Verheyden et al., 2004). While evergreen species in temperate climates may rely less on stored carbon for EW formation (Castagneri et al., 2018; Soudant et al., 2016), Mediterranean species often depend on carbon reserves throughout the growing season (Castagneri et al., 2018).

Arbequina and Arbosana exhibited more isohydric behavior, closing stomata to conserve water and consequently reducing ^13^C discrimination, resulting in higher δ^13^C values (Farquhar et al., 1989). During periods of high photosynthesis and growth in spring and summer, carbon is rapidly transferred from leaves to the cambium, influencing EW isotope composition (Gessler et al., 2014).

In contrast, Koroneiki appeared more anisohydric, maintaining higher stomatal conductance and relying on carbon reserves accumulated during the previous autumn and winter for spring growth (Kagawa et al., 2006; Soudant et al., 2016). Its δ^13^C values correlated with the previous winter’s precipitation, suggesting a distinct growth strategy compared to the other cultivars.

Future studies should conduct detailed xylogenesis analyses on olive trees to link specific portions of tree rings with precise seasonal windows. This would allow for a more refined understanding of the relationships between xylem functional traits derived and the environmental conditions present during wood formation.

## 5 Conclusion

This study provides new insights into the adaptive responses of three olive cultivars (Arbequina, Arbosana, and Koroneiki) to climatic variability, based on dendroecological, anatomical, and high-resolution δ^13^C analyses.

Koroneiki exhibited the most stable growth and hydraulic performance under fluctuating conditions, characterized by higher VD and lower δ¹³C variability compared to Arbequina and Arbosana. These traits indicate an anisohydric strategy, relying on carbon reserves and maintaining consistent water transport, making Koroneiki better suited to regions facing prolonged drought. In contrast, Arbequina and Arbosana demonstrated isohydric behavior, with greater sensitivity to precipitation and temperature fluctuations, reflected by higher δ¹³C variability and more reactive stomatal regulation.

Our findings highlight the superior drought resilience of Koroneiki and its potential for higher fruit production under future climate change scenarios. These results can guide olive cultivation practices in regions increasingly affected by climate variability and water scarcity.

Overall, we reveal distinct cultivar-specific strategies in growth, hydraulic architecture, and physiological responses to seasonal environmental stressors, with significant implications for olive productivity under climate change.

## Data Availability

The datasets presented in this article are not readily available because All the datasets in the work will be made available upon request. Requests to access the datasets should be directed to silvia.portarena@cnr.it.
